# Si Miao San Attenuates Inflammation and Oxidative Stress in Rats with CIA via the Modulation of the Nrf2/ARE/PTEN Pathway

**DOI:** 10.1155/2021/2843623

**Published:** 2021-02-10

**Authors:** Pan Shen, Yao Huang, Xin Ba, Weiji Lin, Kai Qin, Hui Wang, Maotao Du, Ying Haung, Yu Wang, Zhe Chen, Shenghao Tu

**Affiliations:** Department of Integrated Chinese Traditional and Western Medicine, Tongji Hospital, Tongji Medical College of Huazhong University of Science and Technology, Wuhan, Hubei 430030, China

## Abstract

**Objective:**

Si Miao San (SMS) is a traditional Chinese formula used in China to treat rheumatic diseases. To date, its mechanism in rheumatoid arthritis (RA) treatment is uncertain. Our study aims to assess the antiarthritic effects of SMS in experimental arthritic rats.

**Materials and Methods:**

SMS (8.63, 4.31, and 2.16 g/kg/day) was orally administered after the first immunization from day 14 to day 53. The effects of SMS on rats with collagen-induced arthritis (CIA) were evaluated by arthritis score and histological assessment. The levels of cytokines and anti-CII antibodies in rat serum were measured by ELISAs. The expression of oxidative stress parameters was detected by biochemical assay kits. The levels of Nrf2, HO-1, NQO1, and PTEN were determined by western blotting.

**Results:**

Medium- and high-dose SMS treatment significantly decreased arthritis scores and alleviated ankle joint histopathology in the rats with CIA. It inhibited the production of IL-6, TNF-*α*, COX-2, and PGE2 in rat serum. SMS also suppressed the expression of anti-CII antibodies IgG1 and IgG2a. Moreover, SMS significantly suppressed the levels of MDA and MPO in the synovial tissues while increasing the levels of SOD and CAT in the rats with CIA. The levels of Nrf2, HO-1, NQO1, and PTEN were upregulated by SMS in rat synovial tissues.

**Conclusions:**

This study demonstrated that SMS effectively alleviated the disease progression of CIA by decreasing the levels of proinflammatory cytokines and reducing oxidative stress damage, as indicated by IL-6, TNF-*α*, COX-2, and PGE2 levels; inhibiting the overproduction of MDA and MPO; and enhancing antioxidant enzymes by upregulating the Nrf2/ARE/PTEN signalling pathway.

## 1. Introduction

Rheumatoid arthritis (RA) is regarded as a serious threat to human health, affecting approximately 0.5–1.0% of people worldwide [1]. The aetiology and pathogenesis of RA have not been completely clarified. It is believed that the main pathogenesis of RA involves multiple inflammatory cascades, leading to persistent synovial hyperplasia and inflammation and cartilage and bone damage [2].

Proinflammatory mediators, including interleukin (IL)-6, TNF-*α*, and prostaglandin E2 (PGE2), have been reported to play essential roles in synovial inflammation, hyperplasia, and joint destruction [3]. In response to these changes in joints, fibroblast-like synovial cells (FLSs) stimulate the production of the chemokine cyclooxygenase-2 (COX-2), which in turn aggravates joint inflammation and injury. Therefore, reducing the expression of proinflammatory cytokines helps to suppress inflammation, prevent joint damage, and delay the progress of structural damage [4]. Other important inflammation modulators in RA are overproduced reactive oxygen species (ROS) and related enzymes that respond to oxidative stresses, such as catalase (CAT), superoxide dismutase (SOD), and malondialdehyde (MDA). Oxidative stress can disturb the balance of pro-oxidants/antioxidants and plays a crucial role in damage and sustained disease progression [5]. Several studies have shown that overproduction of ROS drives intracellular signalling that promotes synovial inflammatory proliferation [6] and is associated with the severity of RA [7]. Additionally, proinflammatory cytokines promote the expression of ROS and nitrogen species and interact to further exacerbate tissue damage. Thus, inhibiting inflammation and alleviating oxidative stress may be effective treatments against RA.

Studies have identified the essential roles of nuclear factor (erythroid-derived 2)-like-2 factor (Nrf2) in the development and progression of RA [8]. Nrf2 is pivotal for the transcriptional induction of phase II detoxifying enzymes, which constitute a main protective mechanism, by interacting with antioxidant response elements (AREs). A line of evidence suggests that the Nrf2/ARE signalling pathway regulates the expression of cytoprotective enzymes, such as haem oxygenase-1 (HO-1) and NAD(P)H:quinone oxidoreductase-1 (NQO1). Nrf2, NQO1, and HO-1 have shown the ability to suppress the migration and apoptosis of inflammatory cells and downregulate proinflammatory cytokines in RA. The Nrf2/ARE signalling pathway is a promising therapeutic target in various disorders correlated with oxidative stress and inflammation, including RA [9]. Phosphatase and tension homologue deleted on chromosome 10 (PTEN) is a particularly sensitive indicator of oxidative stress that also exerts anti-inflammatory and antiproliferative activities by regulating the activation of PI3-kinase and Nrf2. It has been reported that PTEN is associated with the survival of FLSs in RA [10].

Currently, treatment for RA is aimed at relieving systemic symptoms by reducing inflammation and joint damage. Nonsteroidal anti-inflammatory drugs (NSAIDs), disease modifying antirheumatic drugs (DMARDs), and bio-DMARDs are widely applied, although they can induce some potentially adverse effects, such as opportunistic infections, gastrointestinal disorders, allergic reactions, and liver damage, with long-term use [11]. The limited effectiveness and side effects of current treatment strategies have led to the demand for the development of novel, low-toxicity, and effective antiarthritic drugs. Natural products offer a new research direction for the management of RA.

Si Miao San (SMS) is a traditional Chinese formula for the treatment of rheumatic diseases, especially arthritis. SMS is composed of Cortex Phellodendri Chinensis (Huang Bai), Semen Coicis (Yi Yiren), Rhizome Atractylodes (Cang Zhu), and Radix Achyranthis Bidentatae (Niu Xi). Previous studies have shown that SMS plays an anti-inflammatory role by inhibiting the activation of the MAPK and LPA-ATX signalling pathways [12]. Moreover, SMS can relieve pain and inhibit joint destruction. To gain understanding on the mechanism of SMS treatment of RA, this study was performed to verify the therapeutic effect of SMS in rats with collagen-induced arthritis (CIA) and to determine the association between SMS and the Nrf2/ARE/PTEN signalling pathway and anti-inflammation and antioxidation in the progression of RA.

## 2. Materials and Methods

### 2.1. Animals and Experimental Arthritis

Sixty healthy male Wistar rats weighing 150 g to 200 g were procured from the Hubei Provincial Laboratory Animal Center. The study was approved by the Ethical Committee of Tongji Hospital, Tongji Medical College, Huazhong University of Science and Technology (TJ-A20170504). The rats were maintained in the Central Animal House of Tongji Hospital (Animal Certificate of Conformity: SCXK (Hubei) 2015-0018) under controlled temperature (22 ± 4°C). The rats had easy access to standard food and water. All procedures were conducted in accordance with the Ethical Committee of Tongji Hospital. Before the experiment, the rats were adaptively fed for 7 days. A model of CIA was designed as described previously [13]. Bovine type II collagen (CII) (Chondrex, USA, 2 mg/mL) was dissolved in equal volumes of complete Freund's adjuvant (Sigma, USA) containing inactivated *Mycobacterium tuberculosis*. On day 0, 50 rats were intradermally injected at the base of the tail and back with 300 *μ*L of a CII emulsion. On day 7, the rats were given a fortified immunization with 300 *μ*L of CII dissolved in equal volumes of Freund's incomplete adjuvant. Rats intradermally injected with an equal volume of saline were used as normal controls.

### 2.2. Arthritis Assessment

The measurement of body weight was monitored, and the arthritis score was obtained every 3 days in a blinded manner. The arthritis symptoms were scored as previously described [14]. All four limbs were scored ranging from 0 to 4 points according to the following standard: 0, no abnormal signs; 1, slight swelling or erythema of either the ankle or midfoot; 2, slight swelling of the ankle and foot; 3, modest swelling and erythema; and 4, severe swelling and erythema.

### 2.3. Si Miao San Treatment

Concentrated granules of SMS, composed of Cortex Phellodendri Chinensis (Z002.701MK02), Rhizome Atractylodes (Z002.545MK02), Radix Achyranthis Bidentatae (Z002.585MK02), and Semen Coicis (Z002.955KR02), were purchased from Beijing Tcmages Pharmaceutical Co., Ltd. The Chinese herbs were extracted and concentrated to produce granules. The process was undertaken according to Good Manufacturing Practice (GMP) for Drugs (Chinese FDA, 2010 Version) to guarantee quality. The efficacy of 1 g of SMS granules was equal to that of 6 g of decoction pieces. On day 14, successfully modelled rats with arthritis scores over 4 were randomly allocated to 5 groups: the CIA, methotrexate (MTX), and SMS at low-dose (S-L), medium-dose (S-M), and high-dose (S-H) groups. SMS granules and MTX were dissolved in double distilled water and fully blended prior to use. The positive drug group was treated with MTX (2 mg/kg) once a week, and the SMS groups were administered corresponding doses of SMS granules (2.16, 4.31, and 8.63 g/kg) every day. Rats in the normal control (Con) and CIA groups were administered an equal volume (15 ml/kg) of double distilled water.

The animals were made comfortable until they were sacrificed with 2% pentobarbital on day 53. Blood, knee synovial tissues, and hind paw ankles were excised from the rats.

### 2.4. Histopathological Assessment

Ankle tissues of the right hind paws were isolated and fixed with 4% formaldehyde for 24 h and subsequently decalcified in a 12% EDTA bone decalcifier for 50 days. Then, the ankle tissues were dehydrated, embedded, and sectioned in slices (4 *μ*m). After the administration of haematoxylin and eosin (H&E) staining, each section was observed by an optical microscope (Olympus, Japan), or images were captured at 200× magnification. The histological changes in arthritic characteristics were evaluated blindly and included synovial hyperplasia, cellular infiltrate, bone and cartilage destruction, and pannus. Histopathological arthritis score criteria were as follows: grade 0, no sign of abnormality; grade 1, mild infiltration of inflammatory cells; grade 2, slight hyperplasia of the synovial joint without cartilage destruction; grade 3, moderate synovial hyperplasia, slight joint injury; grade 4, severe synovial inflammation and hyperplasia, moderate joint injury; grade 5, severe cellular infiltration and joint injury [15].

### 2.5. Cytokine and Anti-CII Antibody Measurements by ELISA

The levels of IL-6 (RA20009), TNF-*α* (RA20005), COX-2 (RA20086), PGE2 (RA20013), and anti-CII antibodies IgG1 (RA20987) and IgG2a (RA26903) in blood serum were determined by commercially available ELISA kits (Bio-Swamp, Shanghai, China). The absorbance of all samples was detected at 450 nm by using a microplate reader (Synergy, USA). Each sample was assayed in duplicate, and equivocal findings were repeated.

### 2.6. Oxidative Stress Analysis

After the administration of SMS or MTX for 39 days, the rats in each group were euthanized. Knee synovial tissues were collected from the joints. SOD activity and MPO (myeloperoxidase), MDA, and CAT levels were measured by the corresponding commercial assay kit (Nanjing Jiancheng, China).

### 2.7. Western Blot Analysis

Synovial tissues were lysed in ice-cold RIPA solution containing protease inhibitor to extract the proteins. Equivalent protein samples (30 *μ*g/well) of rat synovial tissues were resolved by 12% SDS-PAGE, blotted onto a polyvinylidene difluoride (PVDF) membrane, and then blocked with 5% fat-free milk for 1 h. The protein blots were incubated overnight with primary antibodies against Nrf2, HO-1, NQO1, and PTEN (1 : 1000, CST, USA) at 4°C. GAPDH acted as an internal control to ensure equal protein loading. After administration with TBST solution, the membranes were incubated with the corresponding secondary antibody for 1 h. The protein intensity was measured using an ImageQuant detection system (Odyssey, USA) and analysed with ImageJ software.

### 2.8. Statistical Analysis

All results are expressed as the means ± SEM. A *P* value <0.05 was considered as statistically significant. Data were analysed by one-way ANOVA. Differences between multiple groups were analysed using Dunnett's multiple comparisons test analysis. Quantitative data analysis was performed using GraphPad Prism (GraphPad Software 8.0, USA).

## 3. Results

### 3.1. Effects of SMS on Arthritis Score and Body Weight

**Figure 1:**
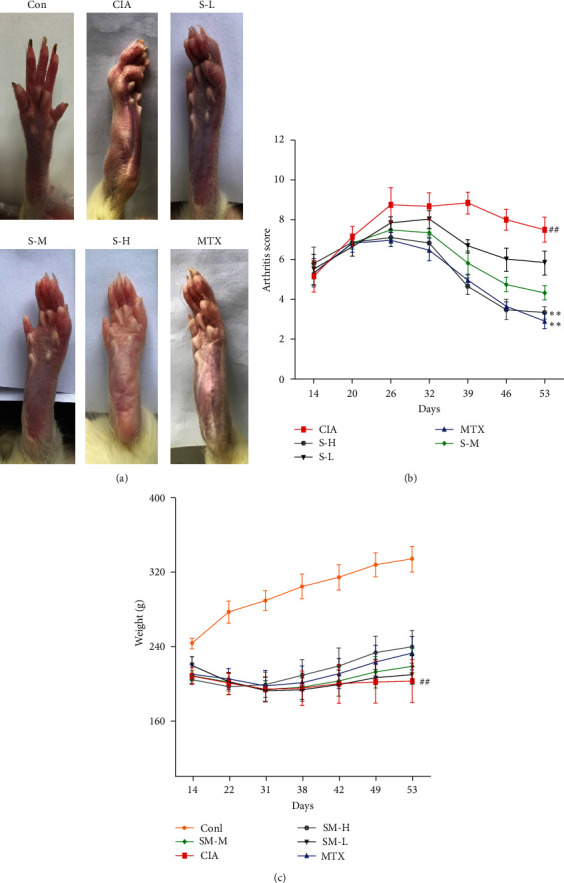
SMS attenuated the severity of disease in the rats with CIA. (a) Paw swelling in rats. (b) Arthritis score of each group. (c) The weights of each group. The data are expressed as the means ± SEM (*n* = 6 rats). ^#^*P* < 0.05, ^##^*P* < 0.01 vs. the control group; ^*∗*^*P* < 0.05, ^*∗∗*^*P* < 0.01 vs. the CIA group.

To determine the positive effects of SMS against RA, a model of CIA was successfully established as a classical RA animal model. Compared to the healthy rats, the animals with CIA were in poor health, exhibiting decreased appetite, decreased muscle strength, and sparse and dull hair. As shown in Figure 1(b), the rats with CIA developed oedema and erythema in the paws, but those in the control group did not exhibit these symptoms (*P* < 0.01) after a second immunization with CII. A plateau was reached between days 26 and 32, after which the arthritis score started to decrease. SMS administration at different doses (4.31 and 8.63 g/kg) significantly reduced the arthritis score compared to saline treatment (*P* < 0.01,  *P* < 0.05). The efficacy of the SMS treatment was comparable to that of MTX, especially at a dose of 8.63 g/kg. Additionally, unimmunized rats showed a continuous increase in body weight. However, the weight of arthritic rats was obviously reduced after the booster immunization and increased slowly compared to healthy rats (*P* < 0.01). SMS and MTX treatment slightly ameliorated the loss of body weight (Figure 1(c), *P* < 0.05).

### 3.2. Effect of SMS on Histopathological Changes in Ankle Tissues

**Figure 2:**
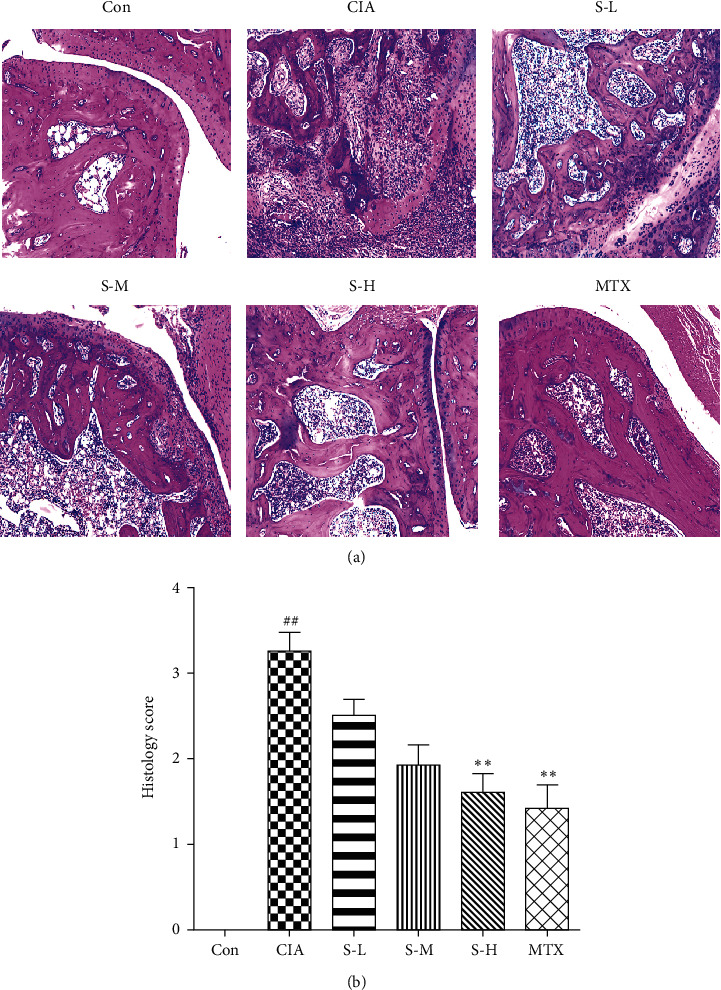
SMS attenuated joint injury in the rats with CIA. (a) Histological analysis of the ankle joint of each group. Original magnification 200×. (b) The histology scores for cellular proliferation, pannus formation, and joint damage were blindly evaluated. The data are expressed as the means ± SEM (*n* = 6 rats). ^#^*P* < 0.05, ^##^*P* < 0.01 vs. the control group; ^*∗*^*P* < 0.05, ^*∗∗*^*P* < 0.01 vs. the CIA group.

A histopathological evaluation of the ankle tissues in the rats with CIA revealed serious cartilage and bone destruction, synovial hyperplasia or congestion, and inflammatory cell infiltration (Figure 2(a)), while the control group rats showed no significant injury in the ankle joints and normal joint space. In contrast, administration of SMS (8.63 g/kg) and MTX showed a prominent reduction in inflammation, synovial cell proliferation, and joint destruction (*P* < 0.05,  *P* < 0.01) (Figure 2(b)).

### 3.3. Effect of SMS on Proinflammatory Cytokines and Anti-CII Antibodies

**Figure 3:**
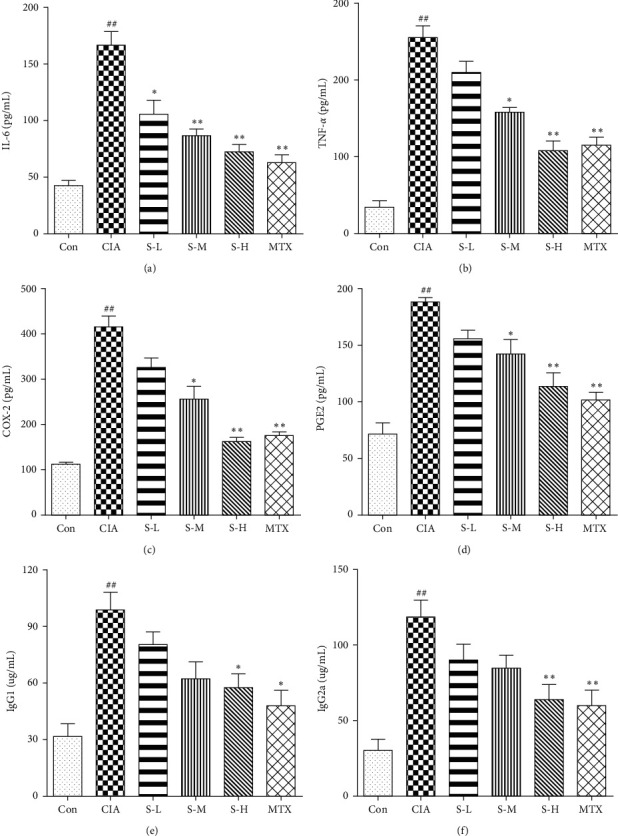
SMS reduced the expression of (a) IL-6, (b) TNF-*α*, (c) COX-2, (d) PGE2, and anti-CII antibodies IgG1 (e) and IgG2a (f), in the serum of CIA rats. All samples were detected by ELISA in duplicate. The data are expressed as the mean ± SEM (*n* = 6 rats). ^#^*P* < 0.05, ^##^*P* < 0.01 vs. the control group; ^*∗*^*P* < 0.05, ^*∗∗*^*P* < 0.01 vs. the CIA group.

As shown in Figure 3, the levels of IL-6, TNF-*α*, COX-2, PGE2, and anti-CII antibodies IgG1 and IgG2a were remarkably upregulated in the serum of the rats with CIA in comparison with the healthy rats (*P* < 0.01), which revealed that CII immunization caused obvious inflammation in the CIA group. However, the levels of proinflammatory cytokines and IgG1 and IgG2a were markedly decreased with the administration of high-dose SMS in CIA animals (*P* < 0.05). The current outcomes showed that SMS significantly inhibited RA-associated inflammation in CIA rats.

### 3.4. Effect of SMS on Oxidative Stress

**Figure 4:**
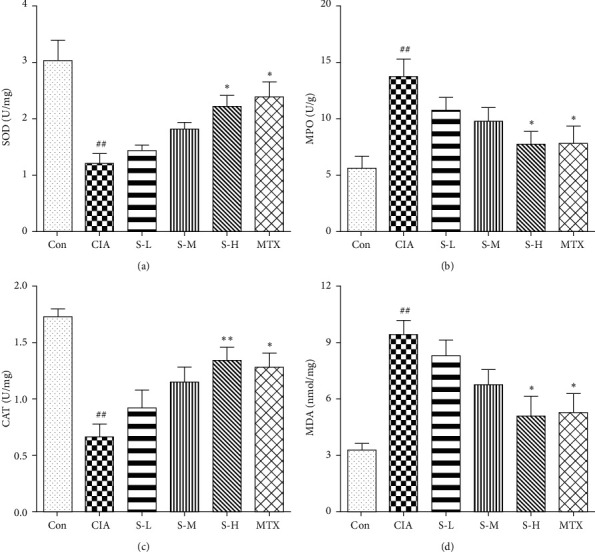
SMS alleviated the expression of (a) SOD, (b) MPO, (c) CAT, and (d) MDA in the synovial tissues of the rats with CIA. The data are expressed as the mean ± SEM (*n* = 6 rats). ^#^*P* < 0.05, ^##^*P* < 0.01 vs. the control group; ^*∗*^*P* < 0.05, ^*∗∗*^*P* < 0.01 vs. the CIA group.

To assess the effect of SMS on oxidative stress, we detected the knee synovial tissue levels of SOD, CTA, MPO, and MDA. As shown in Figures 4(a)–4(d), the levels of MDA and MPO were significantly upregulated in the rats with CIA (*P* < 0.01), while the levels of CAT and SOD were significantly decreased (*P* < 0.01). In contrast, treatment with high-dose SMS significantly suppressed MDA and MPO production and upregulated SOD and CAT levels in the arthritic rats (*P* < 0.05,  *P* < 0.01). The results indicated that SMS might suppress RA activity by alleviating oxidative stress in the CIA model.

### 3.5. Effect of SMS on the Nrf2/ARE/PTEN Pathway

**Figure 5:**
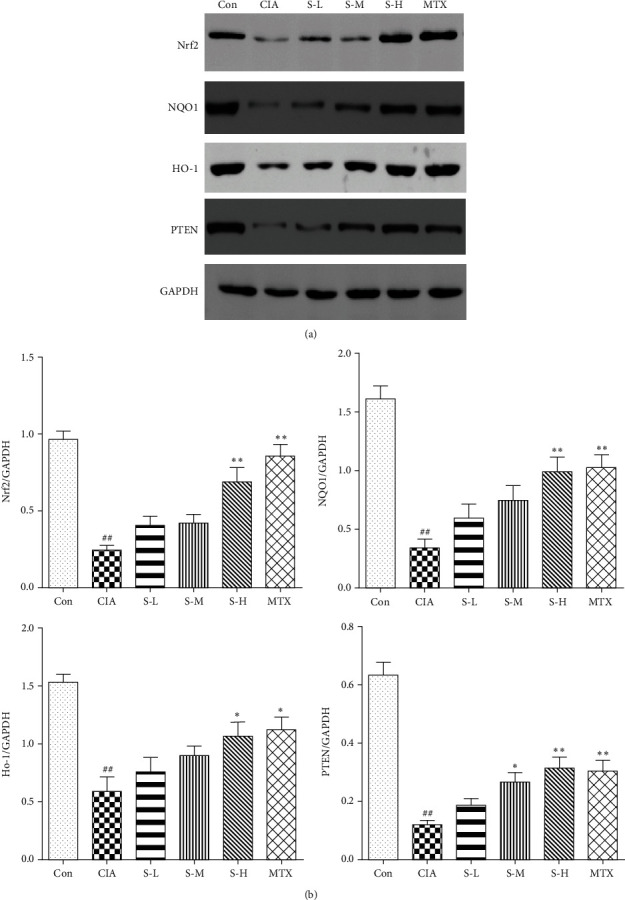
SMS regulated the levels of Nrf2, HO-1, NQO1, and PTEN in the rats with CIA. (a) Representative bands in the western blot for each group. (b) Semiquantitative assessment of the bands in each group. The data are expressed as the mean ± SEM (*n* = 6 rats). ^#^*P* < 0.05, ^##^*P* < 0.01 vs. the control group; ^*∗*^*P* < 0.05, ^*∗∗*^*P* < 0.01 vs. the CIA group.

To further investigate the possible mechanism of SMS action in the rats with CIA, the levels of Nrf2, HO-1, NQO1, and PTEN were determined by western blotting. As shown in Figure 5, the levels of Nrf2, HO-1, NQO1, and PTEN were downregulated in the rats with CIA (*P* < 0.01), while administration of SMS efficiently increased the protein levels in the synovial tissues (*P* < 0.05,  *P* < 0.01). This outcome might suggest that Nrf2/ARE/PTEN signalling participates in the pathogenesis of CIA.

## 4. Discussion

RA is a severe refractory disease characterized by pain, stiffness, and restricted joint movement caused by synovitis, cartilage degradation, and bone destruction [16]. In recent years, several studies have shown the potential antiarthritic effect of natural products with low toxicity [17–19]. An increasing number of researchers are interested in searching for candidate agents for RA treatment from Chinese herbal medicine. As a traditional Chinese medicine, SMS has been applied as a rheumatic disorder treatment that has caused minimal side effects in long-term clinical practice. However, few studies have been performed to investigate the underlying mechanism and pharmacological function of SMS in alleviating RA. CIA is one of the most commonly used models for RA, which shares several characteristics with human RA, such as weight loss and inflammatory cell infiltration in synovial tissues with joint destruction. In the present study, we successfully established a model of CIA in rats and investigated the antiarthritic efficacy of SMS in the rat models. SMS markedly inhibited disease progression, as shown by a decrease in the arthritis score. Histological assessment also revealed that SMS treatment alleviated cellular infiltration, synovial proliferation, and joint damage in the rats with CIA. These changes might be related to anti-inflammatory and antioxidant stress effects through the activation of the Nrf2/ARE/PTEN signalling pathway.

Increasing evidence has revealed that cytokines regulate inflammatory processes in the pathogenesis of RA, especially some proinflammatory cytokines, and play essential roles in synovial inflammation and synovial hyperplasia, which subsequently lead to the cartilage and bone destruction of RA [4, 20, 21]. Proinflammatory cytokines can also intensify inflammation by activating FLSs and osteoclasts and stimulating the production of some inflammatory mediators, such as COX-2, PGE2, and chemokines [22]. It has been suggested that IL-6 levels in serum and synovial fluid in patients are significantly upregulated [23]. In the present study, we confirmed the severity of the rat model of CIA through the increased expression of IL-6, TNF-*α*, COX-2, and PGE2. After treatment with SMS, the TNF-*α*, IL-6, COX-2, and PGE2 levels declined significantly in the rats with CIA. Therefore, it is possible to infer that SMS exerts potential anti-inflammatory effects partially by inhibiting proinflammatory cytokine release in vivo in rats with CIA, thus protecting rats against RA injury.

Oxidative stress also participates in prolonging the inflammation during the pathogenesis of RA and has been described as a biomarker of the severity of RA disease [24]. The development of oxidative stress is related to an imbalance between antioxidants and pro-oxidants. Under oxidative stress, lipid peroxidation changes the permeability of the cell membrane, impairing the activity of membrane-bound enzymes, thereby activating antioxidant enzymes. Excessive oxidative stress has been reported in RA patients and animal models and is accompanied by the overproduction of ROS and MDA and the downregulation of GSH-Px, CAT, and SOD [25, 26], suggesting that MDA is likely to be a critical factor of oxidative damage, while GSH-Px, CAT, and SOD are described as antioxidants. These antioxidant enzymes regulate toxic free radicals; therefore, the reduction in enzymes typically causes a worsening of the disease [27]. MPO, as a marker for neutrophil content, is released from stimulated granulocytes in the damaged area, and its activity is related to the infiltration and activation of polymorphonuclear leukocytes in the inflamed tissue [28, 29]. Moreover, oxidative stress has been found to cause the overproduction of proinflammatory cytokines, especially TNF-*α*. These cytokines also promote the expression of ROS, synergistically aggravating inflammation, the FLS proliferative response, and injury to tissues [30, 31]. Our study found that SMS significantly increased the levels of SOD and CAT and significantly reduced the levels of MPO and MDA in the rats with CIA. Therefore, the antioxidant effects of SMS via the upregulation of antioxidant enzymes had an antiarthritic effect on the rats with CIA. In addition, this antioxidant effect might have also been involved in the inhibited recruitment and infiltration of neutrophils in the joints, thereby protecting the joints.

Nrf2 is mainly involved in regulating the expression of various cellular protective factors and antioxidant enzymes, including HO-1 and NQO1 [32]. Several studies have supported the notion that the Nrf2-mediated pathway regulates not only oxidative stress but also inflammation, immune disorders, hyperproliferation of FLSs, and cartilage and bone metabolism in RA and animal models [33–36]. Moreover, Nrf2 deficiency significantly enhanced oxidative stress, upregulated the expression of COX-2, TNF-*α*, and IL-6, and aggravated joint injury in RA models, which may indicate that it is a potential target for the treatment of inflammatory arthritis [37]. HO-1 is an enzyme that is produced by the degradation of haem to biliverdin and carbon monoxide (CO), which mediates anti-inflammatory, antioxidant, and immune-modulatory effects [38]. The expression of HO-1 is upregulated during increased oxidative stress and in response to proinflammatory cytokines. HO-1 significantly decreased the levels of TNF-*α* and IL-6 and downregulated the protein expression of PGE2 and COX-2 in FLSs in RA [39]. NQO1, another antioxidant enzyme, also participates in the antioxidative stress response, which may play a defensive role in inflammatory diseases [40]. Indeed, the expression of the Nrf2/ARE pathway in RA is associated with disease activity, which may also be related to the apoptosis of macrophages, lymphocytes, and other cells, contributing to the occurrence of RA. PTEN is a sensitive biomarker in oxidative stress [41]. Studies have shown that PTEN exerts anti-inflammatory, antiproliferation, and antioxidative stress by suppressing the activation of the PI3-kinase/AKT pathway. The downregulation of PTEN caused continuous activation of AKT and increased levels of ROS [42]. Moreover, activation of the PTEN target AKT increased Nrf2 nuclear localization, further affecting the activation of Nrf2, facilitating the production of NQO1 and HO-1, and enhancing antioxidative stress [43]. The stabilization of Nrf2 is related to the degree of cell oxidative damage, with cells resist in various apoptotic stimuli in a PTEN-dependent manner [44]. Recent research found that PTEN prominently decreased the arthritis and histology scores and reduced the levels of proinflammatory cytokines in CIA, and the inhibition of PTEN expression in the FLSs promoted their proliferation and migration [45]. PTEN is closely related to the Nrf2 pathway and its mediated inflammatory and oxidative stress responses. PTEN may interact with components in multiple pathways (such as PI3-kinase/AKT) to promote the continued progression of RA; further studies are needed. In the present study, the protein levels of Nrf2, HO-1, NQO1, and PTEN were reduced in the rats with CIA; however, SMS significantly upregulated these proteins. Therefore, these findings indicated that SMS inhibited inflammation and oxidative stress by activating the Nrf2/ARE/PTEN pathway.

## 5. Conclusion

In summary, our study showed that the oral administration of SMS significantly alleviated the symptoms of arthritis and decreased the progression of CIA by suppressing oxidative stress damage and regulating proinflammatory mediators, including the reduced production of MDA and MPO, enhanced SOD and CTA levels, and decreased IL-6, TNF-*α*, COX-2, and PGE2 levels. We also found that SMS upregulated the Nrf2/ARE/PTEN pathway to prohibit inflammation and oxidative stress. All results indicate that SMS has a therapeutic effect for the treatment of RA and that more studies should be carried out to analyse the possible molecular mechanisms.

## Data Availability

The data used to support the findings of this study are available from the corresponding author upon request.
